# Viability PCR shows that non-ocular surfaces could contribute to transmission of *Chlamydia trachomatis* infection in trachoma

**DOI:** 10.1371/journal.pntd.0008449

**Published:** 2020-07-15

**Authors:** Bart Versteeg, Hristina Vasileva, Joanna Houghton, Anna Last, Oumer Shafi Abdurahman, Virginia Sarah, David Macleod, Anthony W. Solomon, Martin J. Holland, Nicholas Thomson, Matthew J. Burton

**Affiliations:** 1 Clinical Research Department, London School of Hygiene & Tropical Medicine, London, United Kingdom; 2 The Fred Hollows Foundation, Addis Ababa, Ethiopia; 3 The Fred Hollows Foundation, London, United Kingdom; 4 Department of Infectious Disease Epidemiology, London School of Hygiene & Tropical Medicine, London, United Kingdom; 5 Parasites and Microbes, Wellcome Trust Sanger Institute, Hinxton, United Kingdom; 6 Moorfields Eye Hospital, London, United Kingdom; RTI International, UNITED STATES

## Abstract

**Background:**

The presence of *Chlamydia trachomatis* (*Ct*) DNA at non-ocular sites suggests that these sites may represent plausible routes of *Ct* transmission in trachoma. However, qPCR cannot discriminate between DNA from viable and non-viable bacteria. Here we use a propodium monoazide based viability PCR to investigate how long *Ct* remains viable at non-ocular sites under laboratory-controlled conditions.

**Methods:**

Cultured *Ct* stocks (strain A2497) were diluted to final concentrations of 1000, 100, 10 and 1 *omcB* copies/*μ*L and applied to plastic, woven mat, cotton cloth and pig skin. Swabs were then systemically collected from each surface and tested for the presence *Ct* DNA using qPCR. If *Ct* DNA was recovered, *Ct* viability was assessed over time by spiking multiple areas of the same surface type with the same final concentrations. Swabs were collected from each surface at 0, 2, 4, 6, 8 and 24 hours after spiking. Viability PCR was used to determine *Ct* viability at each timepoint.

**Results:**

We were able to detect *Ct* DNA on all surfaces except the woven mat. Total *Ct* DNA remained detectable and stable over 24 hours for all concentrations applied to plastic, pig skin and cotton cloth. The amount of viable *Ct* decreased over time. For plastic and skin surfaces, only those where concentrations of 100 or 1000 *omcB* copies/*μ*L were applied still had viable loads detectable after 24 hours. Cotton cloth showed a more rapid decrease and only those where concentrations of 1000 *omcB* copies/*μ*L were applied still had viable DNA detectable after 24 hours.

**Conclusion:**

Plastic, cotton cloth and skin may contribute to transmission of the *Ct* strains that cause trachoma, by acting as sites where reservoirs of bacteria are deposited and later collected and transferred mechanically into previously uninfected eyes.

## Author summary

Trachoma elimination efforts are hampered by limited understanding of *Ct* transmission routes. We have recently demonstrated the presence of *Ct* DNA at non-ocular sites in individuals living in households in Ethiopia where at least one resident had an ocular *Ct* infection detectable by quantitative PCR (qPCR). *Ct* DNA was most frequently detected on faces, hands and clothing, being found in such locations in 10–16% of samples tested. However, qPCR cannot discriminate between DNA from viable and non-viable organisms, and potentially misinform our understanding of *Ct* transmission routes. In this study, we used a propidium monoazide based viability PCR to investigate how long *Ct* remains viable on non-ocular sites by spiking different surfaces including pig skin, plastic and cotton cloth. These surfaces mimic non-ocular sites previously found to be positive for *Ct* DNA using standard qPCR. The results of our study show that viable *Ct* DNA could be recovered from plastic, cotton cloth and skin surfaces for up to 24 hours suggesting that these surfaces a role in ocular *Ct* transmission.

## Introduction

Trachoma, a neglected tropical disease, remains the most common infectious cause of blindness globally, affecting some of the world’s poorest people [1]. Trachoma is caused by repeated ocular infection with ocular strains of the bacterium *Chlamydia trachomatis* (*Ct*). In trachoma-endemic populations, infection is most common in children and is associated with clinical signs of inflammation in the conjunctiva. Chronic inflammation results in immunologically mediated conjunctival scarring and may lead to in-turned eyelashes scratching the eye. Eventually, in some individuals, sight is lost from irreversible corneal opacification [1].

Trachoma elimination efforts are hampered by limited understanding of *Ct* transmission routes and their relative importance. Transmission of ocular *Ct* from infected to uninfected individuals is hypothesised to occur directly through close contact or indirectly on eye-seeking flies and fomites (e.g. face cloths, towels and items of clothing) [1–8]. Using quantitative PCR (qPCR), we have recently tested ocular swabs from 1220 individuals in 247 households living in Ethiopia and ocular *Ct* was detected in 2% of all ages (median *omcB* load 198.6 copies/*μ*L (inter quartile range 23.2–3189.1 copies/*μ*L)) [9]. Moreover, we demonstrated the presence of *Ct* DNA at non-ocular sites in individuals living in these households in Ethiopia where at least one resident had an ocular *Ct* infection detectable qPCR. In these households, *Ct* DNA was most frequently detected on faces, hands and clothing, being found in such locations in 10–16% of samples tested [9]. The presence of *Ct* DNA at non-ocular sites suggests that these sites may contribute to routes of transmission. However, qPCR cannot discriminate between DNA from viable and non-viable organisms [10]. Nucleic acid amplification of non-viable *Ct* could therefore potentially misinform our understanding of *Ct* transmission routes. The assessment of *Ct* viability is essential to gain more insight into transmission processes.

Traditionally, cell culture is the gold standard for the assessment of *Ct* viability, but the sensitivity of culture compared to RNA- or DNA-based nucleic acid amplification tests is low, varying in head-to-head comparisons from 20–83% [11–16]. One promising method to overcome this problem with *Ct* diagnostics is viability PCR which uses propidium monoazide (PMA) as a sample pre-treatment before performing PCR, as recently described by Janssen et al [17, 18]. PMA irreversibly crosslinks with DNA from membrane-impaired (non-viable) bacteria, and by occupying potential primer binding sites, makes it unavailable for amplification and detection by PCR. It has no effect on DNA in bacteria in which the cell membrane is intact, thus only allowing amplification of viable organisms. Viability PCR can therefore improve our understanding of *Ct* transmission by differentiating between DNA from viable and non-viable organisms at non-ocular sites.

Here we use viability PCR to investigate how long *Ct* remains viable on non-ocular sites by spiking different surfaces. We used pig skin to mimic human skin since it is similar to human skin in its histologic structure [19–21]. In addition, we used plastic and cloth that mimic other non-ocular sites previously found to be positive for *Ct* DNA using standard qPCR. The experiments presented in this paper therefore provide further insight into whether these sites contribute to transmission routes in trachoma-endemic communities.

## Methods

### *Chlamydia trachomatis* culture

Human Epithelial type-2 (HEp-2) cells were cultured in 6-well plates (Corning) in standard culture medium consisting of Minimum Essential Medium (MEM; Life Technologies) supplemented with 10% fetal bovine serum (Lonza Bio Science) and 4.5 g/L glucose (Lonza Bio Science) at 37°C in air containing 5% CO_2_. For subcultures, cells were detached with 0.05% trypsin/EDTA (Life technologies).

For infection, ocular *Ct* serovar A strain (strain A2497 [22]) was added to a monolayer of HEp-2 cells at a multiplicity of infection of 1 in the presence of medium supplemented with 10% fetal bovine serum, 4.5 g/L glucose, 2.5 μg/ml amphotericin B (Life technologies) and 20 μg/mL gentamicin (Gibco). Infection was completed by centrifugation at 1800 rpm for 1h at 37°C, and infected cells were incubated at 37°C in air containing 5% CO_2_ for 2 hours. Following this, the medium was replaced with standard culture medium as described above and cells were cultured for another 48–72 hours at 37°C in air containing 5% CO_2_.

HEp-2 cells were then detached using 0.05% trypsin/EDTA-solution (Life technologies) and lysed to release *Ct* elementary bodies (EBs) by sonicating the cells twice for 12 seconds at 80W. Cells were pelleted down at 3800RPM for 10 minutes and resuspended in 0.2M sucrose-phosphate (2SP)-based transport medium containing 0.0125 g/L streptomycin (Generon), 0.0125 g/L vancomycin (Bertin pharma) and 0.625 μg/mL amphotericin B (Life Technologies). A droplet digital PCR (ddPCR) assay was performed as described elsewhere [23, 24] to estimate the number of *Ct* genome (*omcB*) and plasmid (*pORF2*) copies in each culture aliquot.

### DNA extraction

DNA from *Ct* culture or collected swabs was extracted using the Biochain Blood and Serum kit (AMS Biotechnology Europe Ltd). For *Ct* culture, DNA was extracted from an 80 μL aliquot of culture solution. Swabs were vortexed in 500μL 2SP at full speed for two minutes; after expressing excess liquid on the side of the tube, the swab was removed and discarded. DNA extraction of all samples was then completed following the manufacturer's recommendations and eluted in 80μL TE-buffer.

### *Chlamydia trachomatis* quantification and load estimation

*Ct* detection was performed using an in-house multiplex quantitative PCR (qPCR) assay targeting the *Ct* chromosomal *omcB* gene and plasmid *pORF2* gene, as previously described [23]. The assay was performed on a 7900HT Fast Real-Time PCR machine (Applied Biosystems) in 384-well format.

SDS 2.4 software (Life Technologies, Paisley, UK) was used for PCR data analysis. Samples were tested in duplicate and classified as positive for *Ct* if amplification of the *omcB* target was detected within 40 cycles (since *Ct* is known to have only one chromosome copy of *omcB*, but variable numbers of plasmid *pORF2*, per bacterium) [25]. *Ct* load was estimated by extrapolation from an eight-step, ten-fold dilution of standards of known concentration; these were tested in duplicate on each plate.

### Viability PCR

Viability PCR samples were split into two aliquots prior to DNA extraction. One of the aliquots was extracted using standard methods described above and the other was pre-treated with PMA (Biotium). A vial of PMA (0.5 mg) was reconstituted in 782μl 20% DMSO to obtain a stock concentration of 1250 μM and subsequently stored in the dark at 4°C. Treatment conditions were optimized, and PMA concentrations were in line with previous studies [17, 18, 26, 27]. PMA stock solution was added to each sample to a final concentration of 50 μM, with resulting mixtures then incubated for 15 minutes in the dark on ice. All samples were subsequently exposed to blue light-emitting diodes (emission wavelength 465 nm; GenIUL Phast Blue) for 15 min. Total DNA was extracted using the Biochain Blood and Serum kit (AMS Biotechnology Europe Ltd) described above.

### Technical validation of viability PCR

*Ct* culture was heat killed at 95°C for 15 min at 300 rpm on an Eppendorf Thermomixer C (Eppendorf) to demonstrate the efficacy of viability PCR to distinguish between DNA from viable and non-viable *Ct*.

### Preparation of spiked surfaces

Since we previously observed a median *omcB* load of 198.6 copies/*μ*L (inter quartile range 23.2–3189.1 copies/*μ*L) in infected individuals using the same *Ct* qPCR assay [9], a cultured *Ct* aliquot was diluted in 2SP to obtain final load concentrations of 1000, 100, 10 and 1 *omcB* copies/*μ*L to reflect a similar *omcB* load range. All dilutions were confirmed by testing 80 *μ*L aliquots of each solution. Dacron swabs (Puritan, Medline Scientific) and pieces of plastic sheet, woven mat, cotton cloth and pig skin of approximately 4x4 cm size were spiked by inoculating 80 *μ*L of each dilution on to each swab or surface and allowing them to dry for 15 min at ambient room temperature (typically 22–25°C).

### *Chlamydia trachomatis* DNA recovery from spiked surfaces

First, we investigated total DNA recovery from each surface. Dacron swabs were pre-moistened in 2SP and systematically rubbed with moderate and consistent pressure across each surface, horizontally and vertically covering an area of 4x4 cm for ten seconds. A swab was collected from each surface into 500*μ*L 2SP directly after the surfaces were spiked with *Ct* culture solution. An 80 *μ*L aliquot of each final concentration was taken and stored in 500 *μ*L 2SP to serve as a positive control. Swabs were immediately processed for DNA extraction and qPCR as described above. If *Ct* DNA was recovered from a surface, *Ct* viability PCR was performed.

### Time series of *Ct* viability on spiked surfaces from which *Ct* DNA was recovered

*Ct* viability was investigated at 0, 2, 4, 6, 8 and 24 hours after spiking for each surface from which *Ct* DNA was recovered. A separate surface was spiked for each time point and each spiked surface sample was swabbed only once. For each time point, an 80 *μ*L aliquot of each final concentration was taken and stored in 500 *μ*L 2SP to serve as a positive control. All samples were immediately split and separately tested using standard quantitative and viability PCRs.

### Statistical analysis

All data were analysed using R version 3.4.2 (R Foundation for Statistical Computing, 2017). *Ct* load data were log_e_-transformed. Error bars in figures represent standard deviations from two independent experiments. Mixed effects linear regression was used with *omcB* log_e_ copies as the outcome, and time as the exposure variable to estimate the decrease in viable *omcB* log_e_ copies per hour. Decline in viable *omcB* log_e_ copies per hour was only estimated up to 8 hours after spiking since the gap between the 8-hour and 24-hour timepoint provided too much uncertainty for an accurate estimate. The estimated proportional reduction in *omcB* copies per hour was obtained by taking the exponential of each estimated *omcB* log_e_ copy reduction per hour.

## Results

### Technical validation of viability PCR

**Fig 1:**
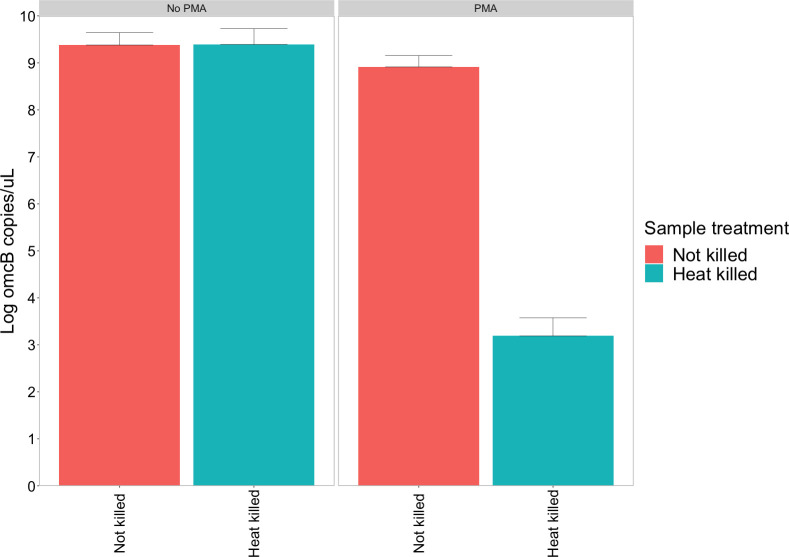
Effect of PMA treatment on viable and non-viable *Chlamydia trachomatis* cultures. Quantitative PCR was performed using primers targeting the single copy *omcB* gene. Error bars represent standard deviations from three independent replicates.

Comparable log_e_ *omcB* loads were observed for the heat-killed and non-heat-killed *Ct* culture aliquots that were not exposed to PMA treatment (log_e_ 9.39 vs 9.38 copies/*μ*L, respectively) (Fig 1). For the non-heat-killed *Ct* aliquots, PMA treatment resulted in a 0.47 log_e_ unit (±0.20) *omcB* load reduction. In contrast, PMA treatment of heat-killed *Ct* aliquots resulted in a 3.49 log_e_ unit (±0.50) *omcB* load reduction relative to the heat-killed *Ct* aliquots without PMA treatment, a 98% reduction. Attempts to culture heat-killed *Ct* aliquots were all negative, providing further evidence that *Ct* were no longer viable after heat killing.

### Immediate *Chlamydia trachomatis* DNA recovery from spiked surfaces

**Fig 2:**
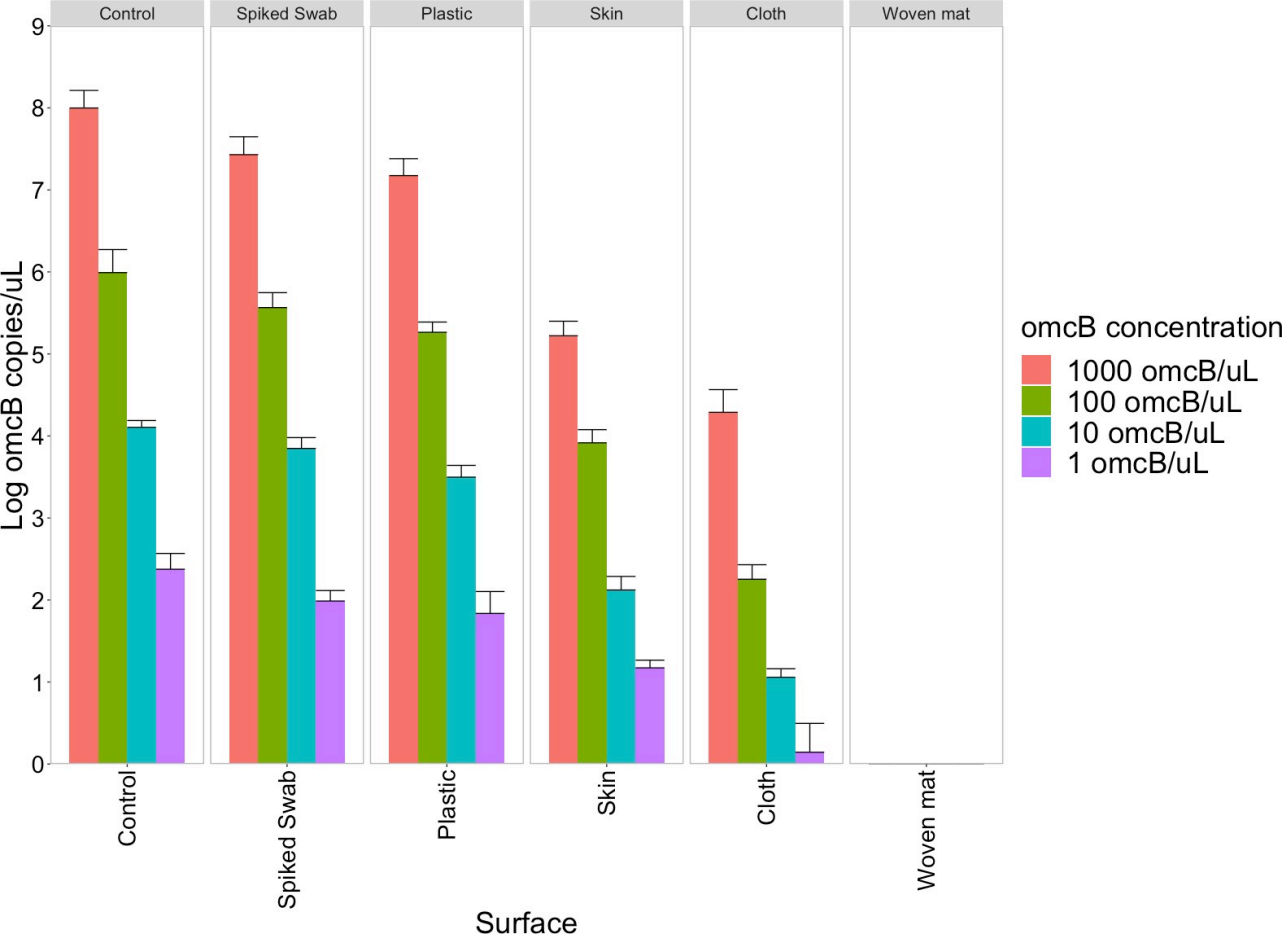
*Chlamydia trachomatis* DNA recovery from spiked surfaces. Quantitative PCR was performed using primers targeting the single copy *omcB* gene. Error bars represent standard deviations from three independent replicates.

*Ct* DNA could be retrieved from all surfaces except the woven mat, although differences were observed in the amount of *Ct* DNA recovery from each surface (Fig 2). The highest percentage recovery was observed for plastic, however, this varied depending on concentrations of *Ct* culture solution used: Lower concentrations of *Ct* culture solution resulted in lower recovery percentages (Table 1).

**Table 1:** *Chlamydia trachomatis* DNA recovery from spiked surfaces.

Surface	Spiked concentration (*omcB* copies/μL)	*omcB* log_e_ load range	Mean log_e_ *omcB* load (sd)	Mean percentage recovery^a^
Spiked swab	1000	7.14–7.72	7.43 (0.22)	100%
	100	5.22–5.76	5.57 (0.18)	100%
	10	3.68–3.99	3.87 (0.15)	100%
	1	1.73–2.05	1.99 (0.13)	100%
Plastic	1000	6.92–7.40	7.17 (0.21)	97%
	100	5.15–5.47	5.26 (0.13)	95%
	10	3.24–3.64	3.85 (0.13)	91%
	1	1.34–2.05	1.84 (0.27)	92%
Skin	1000	5.06–5.38	5.22 (0.18)	70%
	100	3.77–4.06	3.92 (0.16)	70%
	10	1.80–2.25	2.12 (0.17)	55%
	1	1.01–1.25	1.17 (0.09)	59%
Cotton cloth	1000	3.95–4.55	4.29 (0.28)	58%
	100	2.05–2.53	2.25 (0.18)	41%
	10	0.90–1.15	1.06 (0.11)	27%
	1	0.00–0.86	0.14 (0.35)	7%
Woven mat	1000	0.00–0.00	0.00 (0.00)	0%
	100	0.00–0.00	0.00 (0.00)	0%
	10	0.00–0.00	0.00 (0.00)	0%
	1	0.00–0.00	0.00 (0.00)	0%

^a^Mean percentage recovery compared to log_e_ *omcB* load detected on spiked swabs.

### *Chlamydia trachomatis* viability on spiked surfaces over time

Since DNA could not be detected from woven mat, we conducted further experiments to examine recovery of viable DNA over time using only plastic, skin and cotton cloth. Surfaces were spiked and viability PCR was conducted at 0, 2, 4, 6, 8 and 24 hours after spiking.

**Fig 3:**
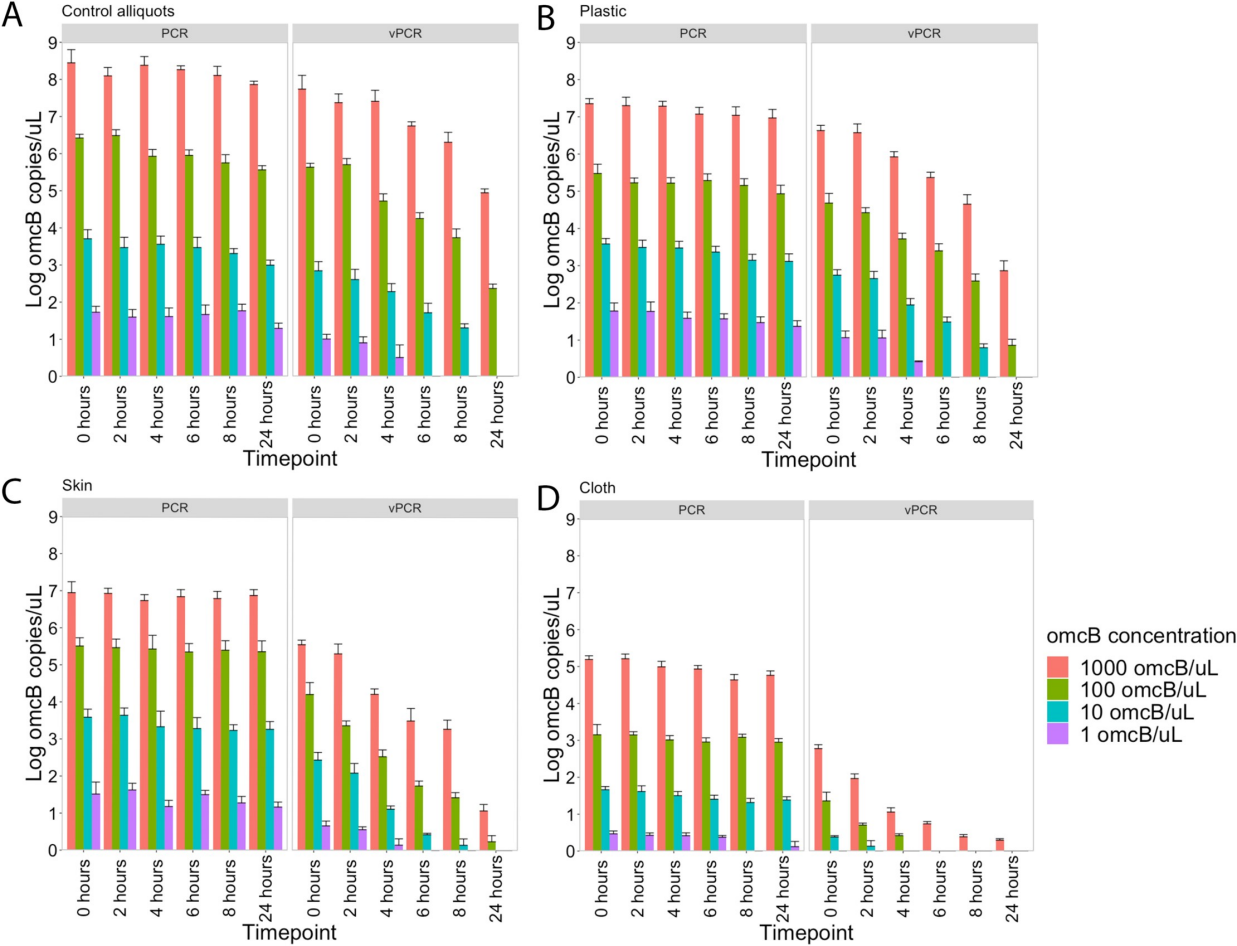
Detection *Chlamydia trachomatis* viability on spiked surfaces over time. Showing (A) detectable viable load in control aliquots, (B) detectable viable load on spiked plastic surface, (C) detectable load on spiked pig skin surface and (D) detectable viable load on spiked cotton cloth surface. Error bars represent standard deviations from two independent replicates.

Total *omcB* (determined by standard qPCR) remained detectable and stable at each timepoint up to 24 hours for all control aliquots (Fig 3A), spiked plastic (Fig 3B), spiked skin (Fig 3C) and spiked cotton cloth (Fig 3D). In contrast, a variable decrease in the proportion of viable *Ct* was observed over time depending on the different surfaces and concentrations used. Control aliquots, plastic and skin gave similar results with only 100 or 1000 *omcB* copies/*μ*L still having detectable viable load after 24 hours, while fluid containing concentrations of up to 100 *omcB* copies/*μ*L left no residual viable load after 4 hrs (1 *omcB* copy/*μ*L) and 8 hrs (10 *omcB* copies/*μ*L). For cotton cloth, a more rapid decrease in detectable viable DNA was observed, with a concentration of 1 *omcB* copy/*μ*L not being detectable at any timepoint. A concentration of 10 *omcB* copies/*μ*L was detectable up to 2 hours and a concentration of 100 *omcB* copies/*μ*L was detectable up to 4 hours. Viable DNA could only be detected up to 24 hours for a concentration of 1000 *omcB* copies/*μ*L. Overall, these results indicate that *Ct* can remain viable at detectable levels on plastic, skin and cotton cloth for up to 24 hours, depending on *Ct* load.

## Estimated decline in viable *Chlamydia trachomatis* per hour

Decline in viable log_e_ *omcB* copies per hour was only estimated for the highest concentration (1000 *omcB* copies/*μ*L) since this was the only concentration that was detectable up to 8 hours on all surfaces. Moreover, we only estimated the decline in viable *Ct* per hour for the first 8 hours since the gap between the 8-hour and 24-hour timepoint introduced too much uncertainty at later time points (Table 2). Decline in viable *Ct* varied per surface with pig skin showing the highest reduction rates: -0.35 log_e_ *omcB* copies per hour (30% reduction in viable load per hour), followed by cotton cloth which showed a decrease in viability of -0.30 log_e_ *omcB* copies per hour (30% per hour) and plastic -0.26 log_e_ *omcB* copies per hour (23% per hour). Control aliquots taken at each timepoint showed a decline in viable *Ct* of -0.22 log_e_ *omcB* copies per hour (20% per hour).

**Table 2:** Estimated decline of viable *Chlamydia trachomatis omcB* copies per hour from spiked surfaces.

	PCR		vPCR	
Surface	Log_e_ reduction^a^	Proportion reduction^a^	Log_e_ reduction^a^	Proportion reduction^a^
Control aliquot	-0.04	4%	-0.22	20%
Plastic	-0.04	4%	-0.26	23%
Pig skin	-0.01	0.8%	-0.35	30%
Cotton cloth	-0.07	7%	-0.30	26%

^a^Reduction refers to the estimated reduction of detectable *omcB* copies per hour.

## Discussion

In this study, viability PCR was used to investigate how long an ocular *Ct* strain remains viable at non-ocular sites in a controlled environment by spiking different surfaces including pig skin, plastic, woven mat and cotton cloth. Using standard qPCR and viability PCR, we demonstrated that viable ocular *Ct* remains detectable on several different surfaces for up to 24 hours. To the best of our knowledge, this is the first study to look at recovery of total and viable ocular *Ct* DNA from different surfaces over time in a reproducibly controlled environment.

We technically validated the use of PMA treatment combined with our *Ct* qPCR assay. Validation was performed by applying viability PCR to a fresh *Ct* culture and *Ct* culture after a heat-kill step. Without PMA treatment, detectable *omcB* loads were similar for cultures with and without heat-killing. When comparing load values before and after PMA treatment of fresh *Ct* culture there was a slight difference in detectable *omcB* load. This difference was most likely caused by the presence of non-viable *Ct* at the start of *Ct* culture that entered HEp-2 cells through centrifugation-assisted inoculation or due to prolonged incubation times (48–72 hours). PMA treatment of *Ct* culture after a heat-kill step significantly reduced qPCR detection of *omcB* load. These results are in line with previous studies validating PMA-based viability PCR for *Ct* [17] and other pathogens [26, 28, 29], all of which demonstrated that PMA treatment of heat-inactivated bacterial cultures resulted in up to a 3–4 log_e_ reduction of detectable target sequences.

*Ct* DNA could be recovered from all surfaces except woven mat. These results are in line with previous work demonstrating that *Ct* DNA could be detected on hands, faces and clothing of individuals and water cans in households where at least one household member had an ocular *Ct* infection detectable by PCR [6, 9, 30, 31]. The lack of detection from woven mat in the present study may have occurred because *Ct* culture in 2SP solution seeped through the material and did not leave sufficient DNA on the surface to allow later recovery. In addition, although we could recover DNA from plastic, skin and cotton cloth, we observed differences in the proportion of DNA we recovered compared to spiked swabs that served as controls. Less DNA was recovered from skin and cotton cloth, which were probably both able to absorb some 2SP solution, than from plastic, on which 2SP solution remained surface-bound.

This is the first study to assess viability of ocular *Ct* over time on different surfaces. Our results demonstrate that *Ct* remains viable on plastic, skin and cotton cloth for up to 24 hours, suggesting that these surfaces could contribute to transmission. However, reduction in viability was dependent on the initial concentrations that were used to spike these surfaces, with lower concentrations becoming non-viable more rapidly (Fig 3). The more rapid decreases in viability for skin and cotton cloth surfaces likely reflects lower DNA recovery from these surfaces. As a result of lower detectable loads directly after spiking, viable loads on these surfaces may become undetectable more rapidly. This may provide some indication of the relative potential of different types of surfaces to act as platforms for onward transmission. Overall, for solutions containing 1000 *omcB* copies/*μ*L, we estimated that the amount of viable *Ct* remaining on surfaces and in control solution declines at 22–30% and 20% per hour, respectively.

Potential limitations of our study should be noted. PMA treatment did not block amplification of all heat-killed organism. Viability PCR may overestimate the proportion of truly viable bacteria, since it assumes viability based on an intact cell membrane [17, 27]. It is possible that a proportion of the DNA we amplified belonged to non-viable organisms that had not yet been affected by loss of membrane integrity. This overestimation may have increased during our time series experiments, depending on the time lag between loss of actual viability and disruption of cell membranes. It is generally believed that cell culture has the highest specificity for assessing *Ct* viability, but unfortunately culture has low sensitivity compared to nucleic acid amplification-based tests, making it a poor method for determining viability in this kind of study [11–16].Our data were obtained in a controlled environment and might therefore be an overestimation of true viability on surfaces in a typical trachoma-endemic household. We cannot rule out that external factors such as exposure to UV light through sunlight, dirt, water or the absence of 2SP transport medium would cause a more rapid decrease in *Ct* viability, or, conversely, that deposition of *Ct* on surfaces within ocular or nasal discharge could prolong survival in the real world. These results can therefore not be simply generalised for affected communities, indicating the need to repeat this study. It is important that swabs collected in such study are stored in appropriate transport medium and that an adequate cold chain can be established to ensure samples are frozen as quickly as possible to prevent any loss of viability.

In conclusion, *Ct* DNA could be recovered from all surfaces except woven mat. Viable *Ct* DNA could be recovered from plastic, cotton cloth and skin surfaces for up to 24 hours. These results suggest that plastic, cotton cloth and skin surfaces may play a role in ocular *Ct* transmission.
